# Efficient reduction of nitric oxide using zirconium phosphide powders synthesized by elemental combination method

**DOI:** 10.1038/s41598-017-13616-5

**Published:** 2017-10-12

**Authors:** Zhen Li, Ning Chen, Jigang Wang, Peishen Li, Ming Guo, Qiang Wang, Chunhong Li, Changzheng Wang, Tao Guo, Shaowei Chen

**Affiliations:** 1 Laboratory for Micro-sized Functional Materials & College of Elementary Education and Department of Chemistry, Capital Normal University, Beijing, 100048 P.R. China; 2 Beijing National Laboratory for Condensed Matter Physics, Institute of Physics, Chinese Academy of Sciences, P.O. Box 603, Beijing, 100190 P.R. China; 3 Beijing Key Laboratory of Functional Materials for Building Structure and Environment Remediation, Beijing University of Civil Engineering and Architecture, Beijing, 100044 P.R. China; 4 Department of Chemistry and Biochemistry, University of California, Santa Cruz, CA 95064 USA

## Abstract

Zirconium phosphide (ZrP) powders were synthesized by elemental combination method via the direct reaction of zirconium powders with red phosphorus, and characterized by XRD, SEM, XPS, XRF, SAED and TEM measurements. The obtained ZrP powders were found to exhibit apparent activity in the ready eliminateion of nitric oxide (NO) via facile redox reactions, and the elimination dynamics was evaluated within the context of various important experimental parameters, such as reaction temperature and gas concentration. At a fixed amount of ZrP powders, an increasing amount of NO would be eliminated with increasing reaction temperature, and complete conversion of NO to N_2_ could be reached in the range of 700 to 800 °C. The addition of NH_3_ also facilitated NO elimination at a fixed reaction temperature. Furthermore, of the products of the elimination process, zirconia (ZrO_2_) powder is a kind of biocompatible material, red phosphorus can be used to produce safety matches, organophosphorous pesticide and phosphor bronze, and the produced N_2_ might be collected and used as a protective gas or be converted into liquid nitrogen for other purposes.

## Introduction

Nitrogen oxides (NO_x_) are harmful gases that give rise to a variety of environmental problems, such as acid rain, photochemical smog, and ozone depletion, which threaten human health to a great extent^1–3^. In the pursuit of a better living environment and with increasingly stringent environmental regulations, NO_x_ emissions have become a research hotspot in the field of environmental science and engineering. NO_x_ storage and reduction (NSR) plays an important role in controlling NO_x_ emissions from automobile sources while permitting operation under predominantly lean-burn conditions^4–6^. So far, the most efficient technique to control NO_x_ emissions from coal-fired power plants and automobiles is the selective catalytic reduction (SCR) of NO (4NO + 4NH_3_ + O_2_ = 4N_2_ + 6H_2_O)^7–11^ and NO_2_ (NO + 2NH_3_ + NO_2_ = 2N_2_ + 3H_2_O) with NH_3_ using various catalysts^12,13^. For SCR of NO by NH_3_, it has been found that NO and NH_3_ are introduced into the reaction vessel at a ratio of 1:1, and a certain amount of O_2_ is essential for the reaction. However, the excessive use of NH_3_ can cause air pollution and the corrosive nature of NH_3_ is also harmful to the experimental apparatus, which may cause secondary pollution. Therefore, it is necessary to reduce the use of NH_3_ for the elimination of NO. Also, the removal of NO_x_ is generally carried out at high temperatures (>600 °C), where many catalysts may lose their activity^14–18^, leading to reduced elimination efficiency of NO_x_. Within this context, it is extremely urgent to find more suitable active species or to develop an efficient, green approach to the elimination of NO_x_ at high temperatures.

Transition metal phosphides (e.g., ZrP, FeP, Ni_2_P, etc.) are known for their hardness and chemical inertness even at elevated temperatures^19–21^. In the past few years, substantial efforts have been devoted to the study of the catalytic properties of transition metal carbides and nitrides^22,23^. Notably, transition metal phosphides possess similar physical properties and even better catalytic activity and selectivity in many reactions such as hydrogen evolution reaction (HER) and oxygen evolution reaction (OER), and have become a new research focus in the field of catalytic materials and water oxidation catalysis. For instance, Ni_2_P has been used as an excellent catalyst precursor for water oxidation catalysts^24^, and FeP as an efficient catalyst for HER^25,26^. Also, there are many studies on the catalytic performance of zirconium phosphate (ZrP_2_O_7_)^27–31^. In contrast, whereas ZrP has been synthesized by a variety of methods in the past decades, such as the reactions of zirconium metal with red phosphorus (elemental combination method)^32,33^, zirconium or zirconium tetrachloride with Ca_3_P_2_^20^, zirconium with PH_3_^33^, or sodium co-reduction of ZrCl_4_ and PCl_3_^34^, there are few reports on the chemical properties of ZrP and, up to date, there is no study of the activity of ZrP towards NO elimination.

In this study, ZrP was synthesized by elemental combination method via the direct reaction of zirconium powders with red phosphorus in a quartz tube. Since the phosphorus atoms in the ZrP are negatively charged (P^3−^) and the nitrogen atoms in the NO are positively charged (N^2+^), ZrP might be used to reduce NO by the reaction, ZrP + 2NO → ZrO_2_ + N_2_ + P. The results showed that the efficiency of NO elimination by ZrP increased with increasing temperature. In addition, it was found that when a small amount of NH_3_ was added to the reaction system, the following reaction could occur, 8ZrP + 22NO + 4NH_3_ → 8ZrO_2_ + 13N_2_ + 8P + 6H_2_O, leading to enhanced efficiency of NO elimination. The experimental results showed that at the ratio of the NH_3_ to NO concentration of 2:11, 0.5 g of ZrP powders was sufficient for the complete reduction of 500 ppm NO gas for up to 14 h at 750 °C. As for the products of the elimination reaction, ZrO_2_ powders can be used as a biocompatible material^35,36^ and catalysts/catalyst support, red phosphorus can be used to produce safety matches and organophosphorous pesticide, and the produced N_2_ may be collected and used as a protective gas or be converted into liquid nitrogen for other purposes. In this study, we not only identified an active species for NO elimination, but also reduced the amount of NH_3_ for NO reduction. The elimination process was of high efficiency and all reaction products could be used for other purposes.

## Results

### Structures of ZrP

**Figure 1:**
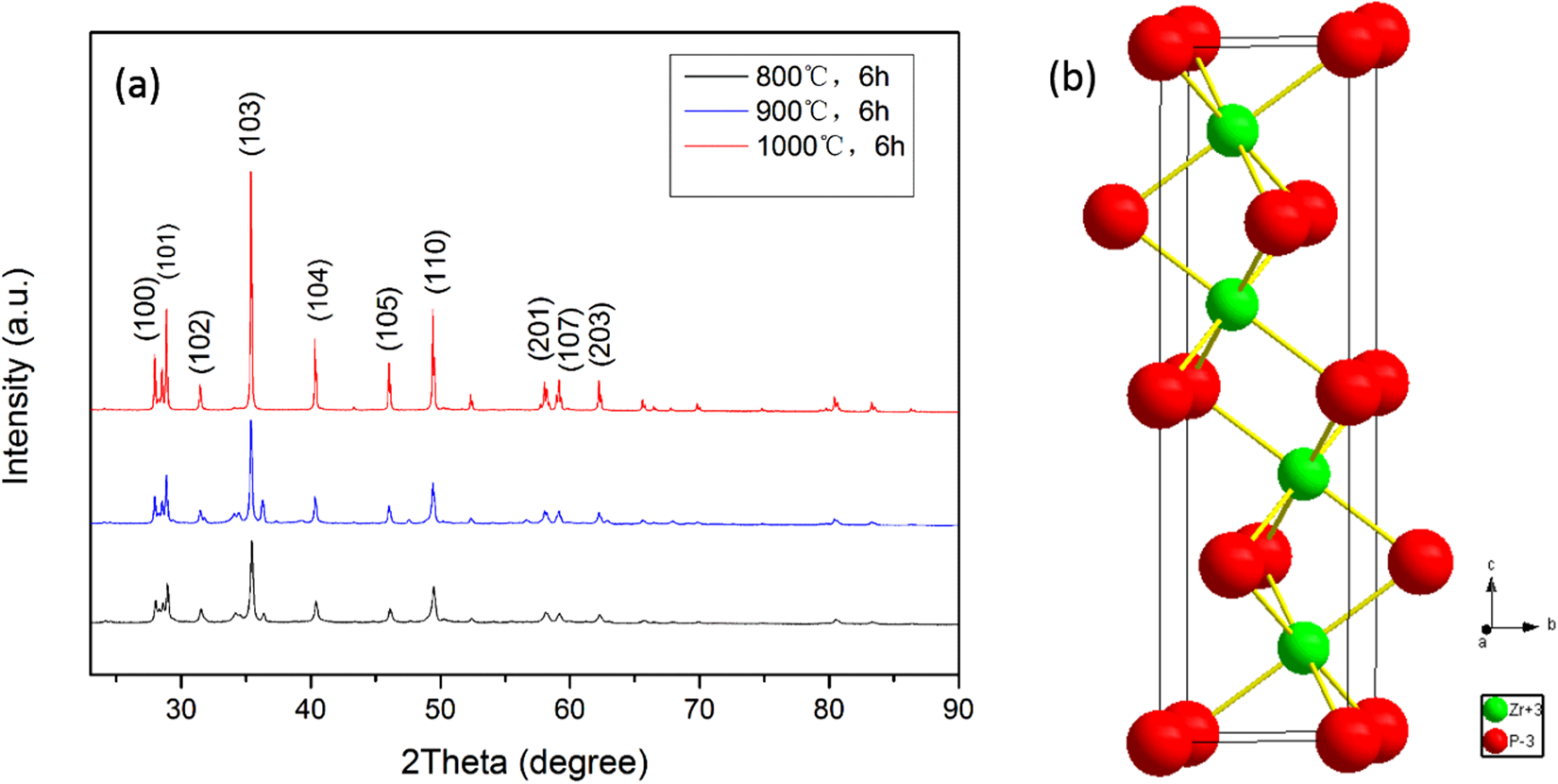
XRD patterns and Crystal structure of the ZrP samples. (**a**) XRD patterns of the ZrP prepared at different temperatures (**b**) Crystal structure of ZrP. The red and green balls represent phosphorus and zirconium atoms, respectively.

The structures of the obtained ZrP were first characterized by X-ray diffraction (XRD) measurements. As shown in the Fig. 1a, XRD measurements of the samples prepared at 800–1000 °C all show a series of distinct diffraction peaks at 27.86°, 28.78°, 31.36°, 35.55°, 40.49°, 46.21°, 49.58°, 58.24°, 59.26° and 62.55°, which can be indexed to the ZrP (100), (101), (102), (103), (104), (105), (110), (201), (107) and (203) lattice planes of the hexagonal phase of ZrP (space group P63/mmc, No. 194). The observed unit cell parameters are a = b = 3.6840 Å, c = 12.5540 Å and α = β = 90°, γ = 49.58°, in good agreement with data in JCPDS No. 016–0034, with the crystal structure of ZrP shown in the Fig. 1b. In addition, from Fig. 1a, it can seen that at higher temperatures, the diffraction peaks become sharper, suggesting increasing crystallinity of the obtained ZrP samples.

### SEM characterization

**Figure 2:**
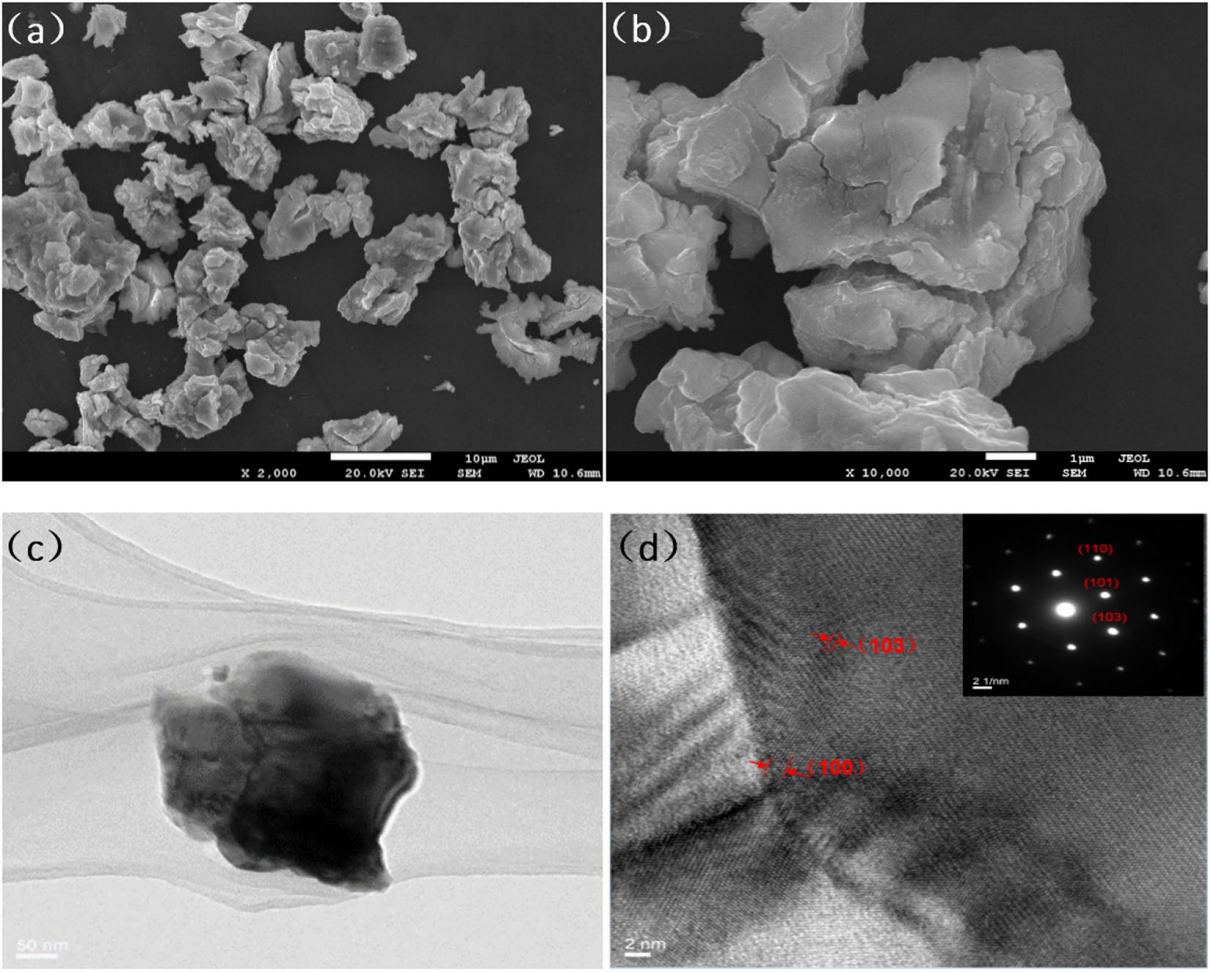
SEM and TEM analyses of the ZrP sample. (**a**) and (**b**) SEM images of the ZrP sample (**c**) TEM images of the as-prepared ZrP (**d**) HRTEM images of the as-prepared ZrP. Inset to (**d**) is the electron-diffraction patterns.

The microstructures of ZrP were then investigated by SEM measurements. From the SEM images (Fig. 2a,b), it can be seen that ZrP was an irregular bulk material.

### TEM characterization

The microstructures of ZrP were also investigated by TEM measurements (Fig. 2c). From the HRTEM image (Fig. 2d), several lattice fringes of 3.2 Å and 2.5 Å can be observed clearly, in good agreement with the crystal planes (100) and (103) of ZrP. In addition, the corresponding SAED patterns (inset to Fig. 2d) can also be assigned to single-crystalline ZrP^42–44^.

### XPS characterization

**Figure 3:**
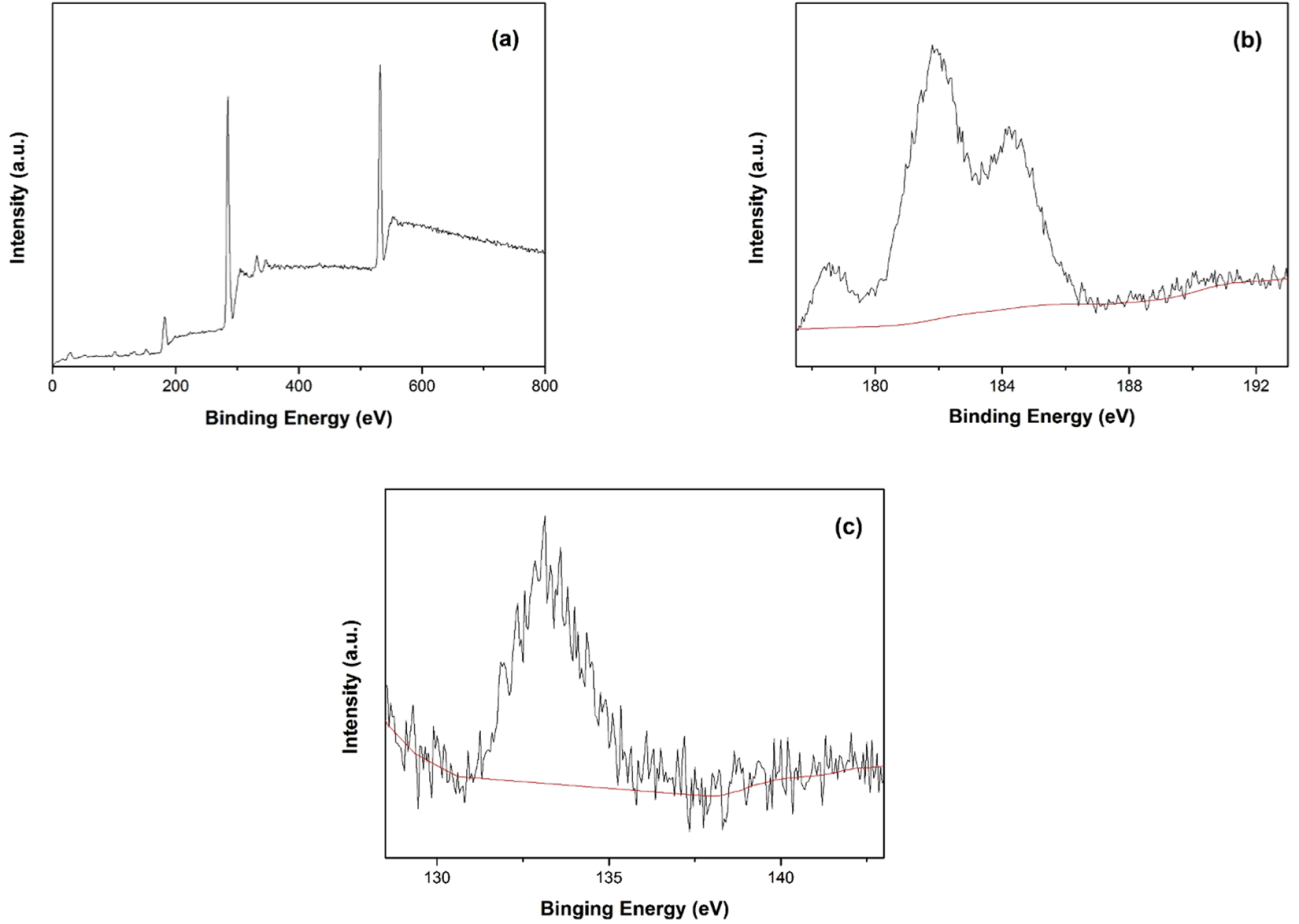
XPS analyses of the ZrP sample. (**a**) survey spectrum (**b**) Zr3d region (**c**) P2p region.

In view of the same diffractions of XRD for ZrP samples prepared at different temperatures (Fig. 1a), only the typical ZrP samples synthesized at 800 °C was selected for XPS, XRF characterization. XPS analyses of the ZrP samples are shown in Fig. 3. The survey spectrum (Fig. 3a) shows that the sample surface consists of zirconium and phosphorus, and based on the integrated peak areas, the mole ratio of Zr/P is estimated to be 1.3:1 (Fig. 3b,c), which is close to that of ZrP. The oxygen may come from surface adsorption. Carbon is also found, which may be from carbon dioxide adsorption.

### XRF characterization

XRF analysis was then carried out to further analyze the composition of the ZrP samples, and the results are summarized in Table 1. It can be seen that for the as-prepared sample, the mole ratio of Zr/P is estimated to be 1.4:1, which is close to 1:1 (when oxygen adsorption on the sample surface was excluded). Yet, after reacting with NO at 700 °C and 750 °C, the relative content of phosphorus in the reaction products diminished with the calculated mole ratio of Zr/P increased from 2.1:1 to 3.6:1. This is consistent with the results shown in Supporting Figure S1.

**Table 1:** Summary of XRF analyses.

Sample	Elemental oxide	wt %
ZrP	ZrO_2_	70.04
	P_2_O_5_	27.32
After reaction with NO at 700 °C	ZrO_2_	77.03
	P_2_O_5_	20.76
After reaction with NO at 750 °C	ZrO_2_	83.81
	P_2_O_5_	13.24

### Elimination of NO

**Figure 4:**
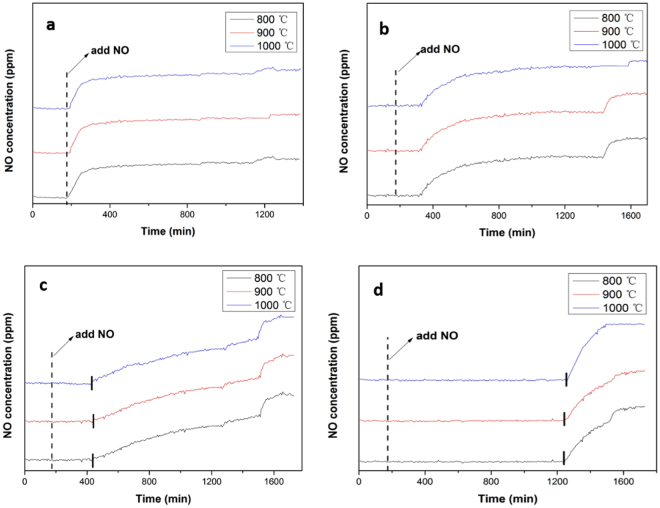
Reductions of NO at 650 °C (**a**), 700 °C (**b**), 750 °C (**c**) and 800 °C (**d**) (three curves in each Figure represented the ZrP samples synthesized at different temperatures, i.e. 800, 900 and 1000 °C, respectively).

**Figure 5:**
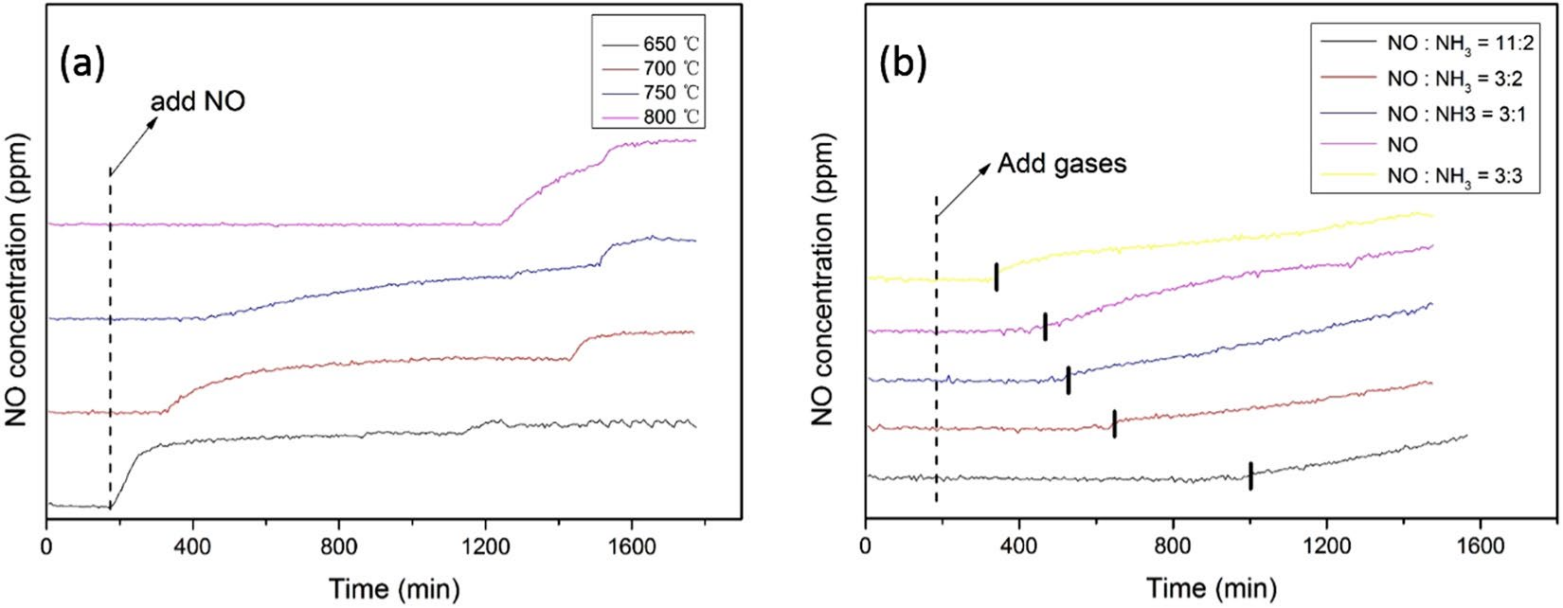
Reaction of NO with ZrP at different temperatures (**a**), Effect of NH_3_ concentration on the elimination of NO (**b**).

Figure 4 showed the reaction of ZrP with NO at different temperatures. Specifically, Fig. 4a shows that when the reaction temperature between ZrP and NO was controlled at 650 °C, it was worth noticing that ZrP samples (prepared at 800 °C, 900 °C, 1000 °C, respectively) would eliminate almost the same amount of NO from three almost identical curves, which indicated that the temperature of synthesis for ZrP had little influence on the elimination of NO at 650 °C. As shown in Fig. 4b–d, when reaction temperatures between ZrP and NO were increased to 700 °C, 750 °C and 800 °C, the elimination amount of NO over ZrP prepared at different temperatures still had no obviously difference. So ZrP samples synthesized at 800 °C was typically selected to the next study of elimination for NO. Figure 5a shows the effect of reaction temperature for the elimination of NO in the temperature range from 650 °C to 800 **°**C. In the course of experiment, a mixture of gases containing 500 ppm NO balanced with N_2_ was introduced into the reactor at the 174th min. It can be seen that at the controlled temperature of 650 °C, after the introduction of NO, the concentration of NO at the outlet increased rapidly and equal to the concentration of NO in the inlet, which means that there was no reaction between NO and ZrP. Yet, when the temperature was raised to 800 °C, no signal of NO was detected at the outlet for 1080 min (18 h), indicating that NO was completely reduced by ZrP in the reactor within this period. At lower temperatures (700 and 750 °C), the period of time where no NO was detected was shorter, suggesting that temperature was an important factor for the elimination of NO. Such a disparity of the reaction activity can also be manifested in XRD measurements of the solids after reaction. From Supporting Figure S1, we can see that when the reaction temperature was controlled at 650 °C, the diffraction patterns of the solids after reaction were consistent with those of the ZrP, as there was no reaction with NO at this temperature. When the reaction temperature was raised to 700 °C, the solids were found to consist of a mixture of ZrP and ZrO_2_, indicating that part of the ZrP was oxidized into ZrO_2_ due to reduction of NO to N_2_. At the even higher temperature of 800 °C, no diffraction pattern of ZrP was detected and only ZrO_2_ diffraction patterns were observed, most likely due to complete consumption of ZrP in the reduction of NO, (ZrP + 2NO = ZrO_2_ + N_2_ + P).

The tests were also carried out to elucidate the influence of NH_3_ addition in NO elimination. As depicted in Fig. 5b, prior to the addition of NH_3_, 0.5 g of ZrP powders can reduce 500 ppm NO gas for 5 h at 750 °C. With the addition of NH_3_ into the reactor where the molar ratio of NH_3_:NO increased from 1:3 to 2:3, the time for the complete conversion of NO into N_2_ was increased from 6 h to 8 h. Yet, too much NH_3_ is not conductive to the elimination of NO. For instance at the mole ratio of NH_3_:NO = 3:3, the time for the complete conversion of NO by ZrP actually decreased to only 3 h, even shorter than that without any ammonia addition at the same condition (~5 h). The optimal NH_3_:NO ratio was identified at 2:11, where 0.5 g of ZrP powders were found to reduce 500 ppm NO gas for up to 14 h at 750 °C. This may be attributed to the following reaction, 8ZrP + 22NO + 4NH_3_ → 8ZrO_2_ + 13N_2_ + 8 P + 6H_2_O. From this reaction, we can see that at a fixed amount of ZrP, the addition of a small amount of NH_3_ could facilitate the reduction of NO. In fact, this elimination process not only reduces the use of NH_3_, but also greatly improves the elimination efficiency of NO as compared to SCR of NO by NH_3_.

### Verification of red phosphorus

After elimination reactions of ZrP with NO, in addition to the product of ZrO_2_, some red brown powders were found adhering to the inner wall at the end of the quartz tube (Supporting Figure S2a), which were collected, dried, and subject to a combustion test. The results showed that the powders burned violently, accompanied by the generation of white smoke (Supporting Figure S2b) and irritating smell, forming a white solid product. These observations were consistent with the combustion of red phosphorus, suggesting red phosphorus as the part of the elimination reaction of NO by ZrP.

## Discussion

ZrP powders were prepared by thermal treatment of zirconium and red phosphorus in an argon atmosphere at controlled temperatures, which exhibited apparent activity in the reductive elimination of NO to N_2_. It was found that the reaction temperature and concentration of NH_3_ were important factors that affected the elimination efficiency of NO by ZrP. The products of the elimination process included ZrO_2_, N_2_, H_2_O (if NH_3_ was added) and red phosphorus. In summary, a new method based on ZrP was developed for the reductive elimination of NO, where the reaction products might be collected and used for other applications.

## Methods

### Material Preparation

All chemicals were purchased from Alfa Aesar and used as received without further purification. To prepare ZrP, zirconium powders were placed in a quartz boat at one end of a quartz tube and the required quantity of 99.99% pure red phosphorus powders were placed at the other end under an atmosphere of purified argon. The temperature was slowly raised (2 °C/min) from room temperature to 800 °C~1000 °C and kept for 6 h^37,38^.

### Material Characterizations

The phase structures and morphologies of the ZrP powders were characterized by X-ray powder diffraction (XRD, Bruker D8 with Cu Kα radiation, λ = 1.54 Å), Scanning electron microscope (SEM, HITACHI, S4800, 15 kV), X-ray photoelectron spectroscopy (XPS, ESCALAB 250 with Al Kα radiation) and X-ray fluorescence (Shimadzu, XRF-1800) measurements. The lattice fringes of the obtained samples and the corresponding selected-area electron diffraction (SAED) patterns were examined by using a high-resolution transmission electron microscope (HRTEM JEOL 2010F, 200 kV).

### Elimination of NO

The elimination of NO was carried out in a fixed-bed quartz tube reactor with an internal diameter of 6 mm^39^. 0.5 g of ZrP powders were sieved with a 40–60 mesh and placed on the quartz wool held in the reactor, and the reactor was heated by a vertical electrical furnace. The total flow rate was 198 mL·min^−1^ (room temperature), the mass of ZrP was 500 mg, and the corresponding gas hourly space velocity (GHSV) was 6 × 10^4^ cm^3^·g^−1^·h^−1^, which was evaluated by the equation (1):

1 $$GHSV=\frac{{q}_{V}}{\pi {{\rm{hr}}}^{{\rm{2}}}},$$

where q_v_ is the total flow rate, h is the height of the reactant in the reactor and r is the radius of the reactor^40^. The feed contained 500 ppm of NO, 500 ppm of NH_3_ (when used), and balance of N_2_. The concentration of NO was continuously detected by a gas chromatographic analyzer equipped with a flame photometric detector (Beijing Beifen-Ruili 3420 A). The NO conversion was calculated according to the equation (2):

2 $$N{O}_{conversion}=\frac{{[NO]}_{in}-{[NO]}_{{\rm{out}}}}{{[NO]}_{in}}\times 100 \% ,$$

where [NO]_in_ indicates the inlet NO concentration at steady state and [NO]_out_ indicates the sum concentration of outlet NO^41^.

### Data Availability

All data generated or analysed during this study are included in this article (and its Supplementary Information files).

## Electronic supplementary material

Supplementary information
