# Supplementary material for: Efficient reduction of nitric oxide using zirconium phosphide powders synthesized by elemental combination method
Source: Sci Rep. 2017 Oct 12;7:13044. doi: 10.1038/s41598-017-13616-5 (PMC5638856; doi:10.1038/s41598-017-13616-5)
Supplement: Supplementary file 1 — Supplementary information [file 41598_2017_13616_MOESM1_ESM.pdf]

Supporting information

Efficient reduction of nitric oxide using zirconium phosphide powders synthesized by elemental combination method

Zhen Li[1], Ning Chen[1], Jigang Wang[1], Peishen Li[1], Ming Guo[1], Qiang Wang[1,*], Chunhong Li[2], Changzheng Wang[3,*], Tao Guo[3], Shaowei Chen[4,*]

[1]Laboratory for Micro-sized Functional Materials & College of Elementary Education and Department of Chemistry, Capital Normal University, Beijing, 100048, P.R. China.

[2]Beijing National Laboratory for Condensed Matter Physics, Institute of Physics, Chinese Academy of Sciences, P.O. Box 603, Beijing 100190, P.R. China.

[3]Beijing Key Laboratory of Functional Materials for Building Structure and Environment Remediation, Beijing University of Civil Engineering and Architecture, Beijing, 100044, P.R. China.

[4]Department of Chemistry and Biochemistry, University of California, Santa Cruz, CA 95064, USA.

[*]Corresponding author. E-mail: qwchem@gmail.com (Q. Wang); changzhwang@163.com (C.Z. Wang); shaowei@ucsc.edu (S.W. Chen).

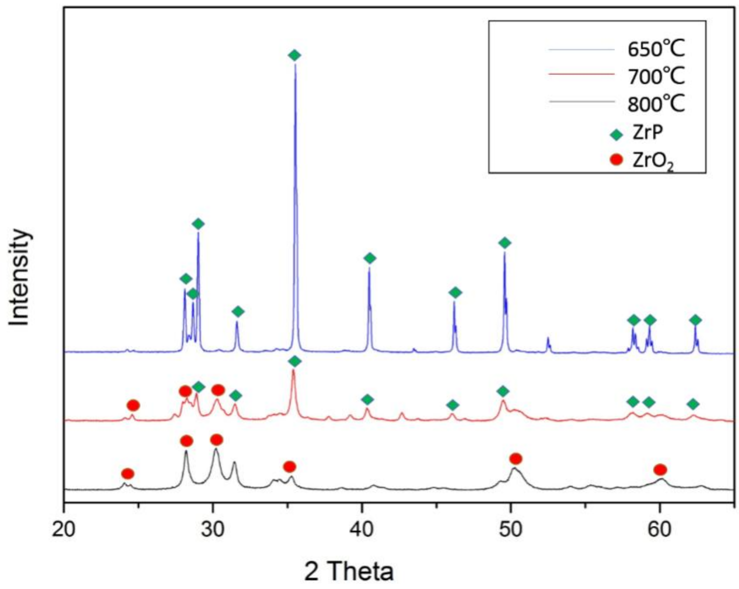

Supporting Figure S1. XRD patterns of the solids after reaction with NO at different temperatures.

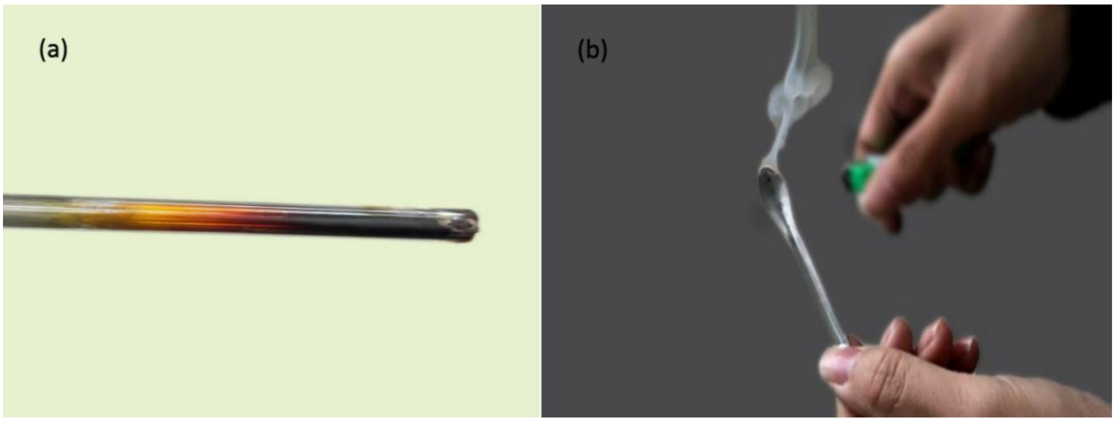

Supporting Figure S2. (a) Product appearance and (b) combustion test of the red component in panel
